# Geospatial analysis of tegumentary leishmaniasis in Rio de Janeiro state, Brazil from 2000 to 2015: Species typing and flow of travelers and migrants with leishmaniasis

**DOI:** 10.1371/journal.pntd.0007748

**Published:** 2019-11-15

**Authors:** Luciana de Freitas Campos Miranda, Raquel da Silva Pacheco, Maria Inês Fernandes Pimentel, Mariza de Matos Salgueiro, Aline Fagundes da Silva, Cíntia Xavier de Mello, Juliana Helena da Silva Barros, Claudia Maria Valete-Rosalino, Maria de Fátima Madeira, Samanta Cristina das Chagas Xavier, Armando de Oliveira Schubach

**Affiliations:** 1 Laboratório de Pesquisa Clínica e Vigilância em Leishmanioses, Instituto Nacional de Infectologia Evandro Chagas, Fundação Oswaldo Cruz, Rio de Janeiro, RJ, Brazil; 2 Laboratório Interdisciplinar de Pesquisas Médicas, Instituto Oswaldo Cruz, Fundação Oswaldo Cruz, Rio de Janeiro, RJ, Brazil; 3 Laboratório de Biologia de Tripanosomatídeos, Instituto Oswaldo Cruz, Fundação Oswaldo Cruz, Rio de Janeiro, RJ, Brazil; Temple University, UNITED STATES

## Abstract

**Background:**

We identified the species of *Leishmania* isolated from traveling and migrant patients attended in a reference center from 2000 to 2015, we performed the georeferencing of these species in Rio de Janeiro (RJ) state and we had knowledge about the human flows between the likely location of infection (LLI) and place of residence (PR) in RJ state, Brazil.

**Methodology/Principal findings:**

This is a retrospective cross-sectional study including 171 patients diagnosed with ATL. Google Maps, OpenStreetMap, and Bing Maps were tools used to georeference LLI and PR. For etiological identification, we used isoenzyme electrophoresis, polymerase chain reaction-restriction fragment length polymorphism (molecular target *hsp70C* with restriction enzymes *Hae*III and *Bst*UI), and sequencing of the internal transcribed spacer of ribosomal DNA. ARCGIS software was used to create maps of the geographic distribution of *Leishmania* species in the state and municipality of RJ, together with flows between the LLI and PR. Isolates from 104 patients were identified as: *L*. *(Viannia) braziliensis* (80.8%), *L*. *(V*.*) naiffi* (7.7%), *L*. *(V*.*) guyanensis* (6.7%), *L*. *(Leishmania) amazonensis* (1%), and genetic variants of *L*. *(V*.*) braziliensis* (3.8%). The flow maps showed that the LLI included 4 countries, 19 Brazilian states, and 18 municipalities of RJ state. The Brazilian states with the highest density of cases were Amazonas (n = 32), Bahia (n = 18), and Ceará (n = 15).

**Conclusions/Significance:**

This work is the first contribution to the knowledge of the routes of *Leishmania* species introduced in RJ state by migrants and travelers patients. *L*. *(V*.*) braziliensis*, *L*. *(V*.*) guyanensis*, *L*. *(V*.*) naiffi*, *L*. *(L*.*) amazonensis*, and genetic variants of *L*. *(V*.*) braziliensis* were identified in RJ state. To determine whether the autochthonous transmission of these imported species is possible it is necessary the adaptation of these species to environmental conditions as well as the presence of reservoirs and phlebotomine vectors in this region.

## Author summary

Leishmaniasis is one of the most important neglected parasitic diseases in humans worldwide. American Tegumentary Leishmaniasis (ATL) is a disease caused by protozoa of the genus *Leishmania*, which are injected into the skin by small insects known by sandflies. Many different *Leishmania* species may cause ATL, which can affect the skin and/or oral and nasal mucosa. In this study, we analyzed flows between the likely location of infection (LLI) and the place of residence (PR) in Rio de Janeiro (RJ) state and identified *Leishmania* species isolated from travelers and migrants attended between 2000 and 2015 in a reference hospital for the care of patients with ATL. This work is the first contribution to knowledge about the routes of *Leishmania* species introduced to RJ state by migrant and traveling patients. In addition to *Leishmania (Viannia) braziliensis*, which was the most common species, other species have also been introduced to RJ state, including *L*. *(V*.*) guyanensis*, *L*. *(V*.*) naiffi*, *L*. *(Leishmania) amazonensis*, and genetic variants of *L*. *(V*.*) braziliensis*. Knowledge of the migratory flow and geographic location of these imported *Leishmania* species may contribute to the surveillance and control of ATL in RJ state, Brazil.

## Introduction

American Tegumentary Leishmaniasis (ATL) is a vector-borne disease, caused by different species of protozoa of the genus *Leishmania* and it is one of the most important infectious diseases worldwide, especially in countries with poor socioeconomic conditions [1]. In Brazil, the main clinical presentations of ATL are cutaneous leishmaniasis (CL), mucosal leishmaniasis (ML), and mucocutaneous leishmaniasis (MCL). CL is the most common clinical form and ML is the most severe form, although it is rarely fatal [2].

Currently, approximately 53 *Leishmania* species have been described worldwide, of which, 31 species are known to be parasites of mammals and 20 species are pathogenic for human beings [3]. In Brazil, seven species of dermotropic *Leishmania* are described: *Leishmania (Leishmania) amazonensis*, *L*. *(Viannia) guyanensis*, *L*. *(V*.*) shawi*, *L*. *(V*.*) naiffi*, *L*. *(V*.*) lainsoni*, *L*. *(V*.*) lindenbergi*, and *L*. *(V*.*) braziliensis*, the species with the highest prevalence. The geographical distribution of ATL is associated with the dispersion of vectors. There are approximately 1,000 valid described species of sandflies in the world of which 530 are known to occur in the Americas [4]. In Rio de Janeiro (RJ) state, the main vector is *Lutzomyia (Nyssomyia) intermedia*, with *Lu*. *migonei* being considered as secondary vector. Other potential vector species were recorded in endemic areas of RJ state, such as *Lu*. *(N*.*) whitmani*, *Lu*. *(N*.*) flaviscutellata*, *Lu*. *(Pintomyia) fischeri* and *Lu*. *(P*.*) pessoai* [5]. Moreover, there are other vectors of some non-endemic species in RJ state. Therefore, if some non-endemic *Leishmania* also exists in the same region, autochthonous transmission is possible.

In Brazil, ATL was initially considered a zoonosis of wild animals, which occasionally affected people in contact with forests, in this case being considered as anthropozoonosis. However, environmental changes caused by human beings have modified the epidemiological profile of leishmaniasis in some regions, where sandflies vectors have adapted to the domestic and peridomiciliary environment and domestic animals also participate in this cycle [6].

Many methods have been described for typing *Leishmania* species in clinical or cultured samples. Multilocus enzyme electrophoresis (MLEE) is the basis of current *Leishmania* taxonomy and is still considered to be the gold standard for parasite typing, despite being a time-consuming method that is only applicable to cultured parasites [7,8]. Using molecular tools such as polymerase chain reaction-restriction fragment length polymorphism (PCR-RFLP) of the gene encoding the heat shock protein hsp70, it is possible to identify *Leishmania* species in samples that are difficult to grow in culture. This target has been used to identify *Leishmania* species from the Old and New World [9,10]. In this technique, the amplicons are subjected to restriction enzyme digestion to discriminate the main species of pathogenic *Leishmania* in Brazil: *L*. *(V*.*) braziliensis*, *L*. *(L*.*) amazonensis*, *L*. *(V*.*) guyanensis*, *L*. *(V*.*) shawi*, *L*. *(V*.*) naiffi*, *L*. *(V*.*) lainsoni*, and *L*. *(L*.*) infantum* [10,11]. Another alternative for taxonomic classification is Sanger sequencing of the internal transcribed spacer of ribosomal DNA (ITS1-rDNA). This target is used in phylogenetic studies and classification of species of the genus *Leishmania* [12–14].

The state of RJ, located on the coast in the Southeast Region of Brazil, is the third most populous state in the country, with about 17 million inhabitants and a large influx of tourists [15]. Between 2001 and 2015, 202,164 cases of ATL were reported in Brazil, of which 1,620 (0.8%) were in Rio de Janeiro (RJ) state [16]. In this state, ATL is caused almost exclusively by *L*. *(V*.*) braziliensis*, although an autochthonous case of diffuse CL caused by *L*. *(L*.*) amazonensis* has been recorded [17] and two cases of human infection with *L*. *(L*.*) infantum*, whose only clinical manifestation was the presence of skin lesions typical of ATL, without visceral involvement [18,19]. Additionally, cases of mixed infection with *L*. *(L*.*) infantum* and *L*. *(V*.*) braziliensis* have been detected in humans and dogs [20–22].

Geographic information systems (GIS) are spatial analysis tools that allow for the integration of host, vector, parasite, and environmental data, to understand the transmission patterns and epidemiology of leishmaniasis, Chagas disease, malaria, dengue, and other infectious diseases, as well as their spatiotemporal distribution [23–26]. The epidemiological complexity of ATL is evidenced by the diversity of *Leishmania* species, reservoirs, and vectors involved in the transmission cycle. The identification and monitoring of territorial units of epidemiological significance, as well as knowledge of the spatial distribution of leishmaniasis cases and the species involved, may help to guide control measures [2].

In recent decades, there has been an increase in the number of imported cases of leishmaniasis, which is associated with increased tourism, military training, and migratory flows from endemic areas [27,28]. Travelers can play an important role in the spread of infectious diseases. The greater the flow of migrants and travelers, the greater the likelihood of introducing infectious diseases into their destination regions. However, in the case of leishmaniasis the spread of the disease is also related to the presence of their respective phlebotomine vectors.

The Evandro Chagas National Institute of Infectious Diseases, Oswaldo Cruz Foundation (NIID/FIOCRUZ) located in Rio de Janeiro municipality is responsible for the care and diagnosis of 25% of the ATL cases reported in RJ state, including about 75% of the more serious cases, such as ML or relapses [29]. In Brazil, the identification of the causative species of ATL is not routinely performed. However, it is believed that in RJ state, ATL is caused almost exclusively by *L*. *(V*.*) braziliensis*. In the present study, we identified the species of *Leishmania* isolated from traveling and migrant patients attended at the NIID/FIOCRUZ reference center from 2000 to 2015, we performed the georeferencing of these species in RJ state and taking into account the human flows between the likely location of infection (LLI) and place of residence (PR) in RJ state, Brazil.

## Methods

### Study design and research participants

This was a retrospective cross-sectional study including 171 patients diagnosed with ATL according to clinical, epidemiological and laboratory criteria, treated at NIID-FIOCRUZ from 2000 to 2015. Clinical and epidemiological criteria included lesions in exposed areas (mainly ulcers with infiltrated borders or infiltrated plaques), not responsive to local or systemic antibiotic therapy, in patients residing or traveling to places where ATL is endemic. The laboratory diagnosis included parasitological examinations (culture, direct and histopathological examination), ELISA, Montenegro skin test or molecular method (kDNA PCR). Direct examination refers to the search for amastigote forms in glass slides of Imprint (compression of the tissue fragment) or scraping procedures of cutaneous lesions.

Patients with an LLI that was different from their PR in RJ state were eligible for this study. The LLI was defined through a careful medical interview, with questions regarding current and previous places of residence, trips for work or leisure reasons, and possible exposures in endemic areas. Migrant or traveling patients with an undetermined LLI and without identified *Leishmania* species were excluded from the study. Medical records were reviewed to obtain patient data, such as age, sex, profession, clinical form, residence address, LLI, and reason for exposure. Regarding the reason for exposure, the patients were separated according to four categories:

➢ Migrants: patients who lived in endemic regions of RJ state, other Brazilian states, or from other countries, who moved to a new residential address in RJ state.
➢ Business travelers: patients residing in RJ state who traveled for work reasons to endemic regions, where they acquired the infection.
➢ Leisure travelers: patients residing in RJ state who traveled for recreation to endemic regions, where they acquired the infection.
➢ Undetermined: patients with more than one reason for exposure.

### Diagnostic and laboratory procedures

In 117 patients, the diagnostic technique used was parasitic isolation in culture, according to protocol registered in https://dx.doi.org/10.17504/protocols.io.22tggen. Briefly, fragments from a cutaneous or mucosal lesion were cultured in Novy-MacNeal-Nicolle medium plus Schneider's Drosophila Medium (Sigma-Aldrich, St. Louis, Missouri, USA) supplemented with 10% fetal bovine serum and antibiotics penicillin and streptomycin. The parasites isolated in culture were cryopreserved in liquid nitrogen (N_2_L).

Growth of promastigote cells was performed in sterile bottles for cell culture until the parasites reached the stationary phase of growth. The total culture volume was centrifuged and the pellet was submitted to three washes in NaCl-EDTA buffer under centrifugation to obtain the parasite mass, which was stored in N_2_L until MLEE could be performed. Samples with reduced growth in culture that did not reach the ideal parasite expansion were submitted to the same washing protocol, and the obtained parasite masses were stored in a freezer at –20°C until DNA extraction to carry out molecular techniques.

In addition to samples with reduced growth in culture, samples that presented variant profiles in one or more enzymatic systems using the MLEE technique were also analyzed by PCR-RFLP, for species confirmation. Sanger sequencing of the ITS1-rDNA was performed only in cases where it was not possible to obtain taxonomic identification by MLEE or PCR-RFLP.

### Etiological identification of *Leishmania* species

#### Multilocus enzyme electrophoresis (MLEE)

Identification of species was determined preferably by MLEE, which is a biochemical characterization technique based on the pH dependent electrophoretic mobility of a predefined set of proteins in a gel. The MLEE was performed on 1% agarose gels supported by GE Healthcare GelBond film (124 x 258 mm), according to previously described procedures [7], using six or seven enzymatic systems: 6PGDH (6-phosphogluconate dehydrogenase, EC 1.1.1.43); GPI (glucose phosphate isomerase, EC 5.3.1.9); NH (nucleotidase, EC 3.2.2.1); G6PDH (glucose-6-phosphate dehydrogenase, EC 1.1.1.49), PGM (phosphoglucomutase, EC 5.4.2.2); ME (malic enzyme, EC 1.1.1.40) or MDH (malate dehydrogenase, EC 1.1.1.37). The MDH enzymatic system was used only in cases with variant profiles in the other enzymatic systems. Isoenzyme electrophoresis was performed with the reference strain of *L*. *(V*.*) braziliensis* (MHOM/BR/1975/ M2903). If any sample presented a profile different to this reference, a new assay was performed with the other reference strains: *L*. *(L*.*) amazonensis* (IFLA/BR/19767/PH8), *L*. *(V*.*) guyanensis* (MHOM/BR/1975/M4147), *L*. *(V*.*) shawi* (MCEB/BR/1984/M8408), *L*. *(V*.*) lainsoni* (MHOM/BR/1981/M6426), *L*. *(V*.*) naiffi* (MDAS/BR/1979/M5533), and *L*. *(L*.*) infantum* (MHOM/BR/1974/PP75). Analysis of gel bands was performed qualitatively, by visual comparison of the sample band profiles with the default reference strains.

#### Polymerase chain reaction (PCR)

DNA extraction from the parasite masses was performed using a DNAzol Reagent kit (Invitrogen), following the manufacturer's recommendations. PCR assays were performed using the primers 5'GGACGAGATCGAGCGCATGGT3' and 5'TCCTTCGACGCCTCCTGGTTG3', to amplify a 234-bp fragment of the gene region encoding hsp70C, as previously described [10]. The amplification products were separated using 2% agarose gel electrophoresis with ethidium bromide (0.5 μg/mL) and visualized under ultraviolet light.

#### Restriction fragment length polymorphism (RFLP)

Amplification products obtained by PCR were digested with two restriction enzymes, *Hae*III (Sigma) and *Bst*UI (Thermo Scientific), following the manufacturer's recommendations. The fragments obtained by enzymatic digestion were separated on a 12.5% polyacrylamide gel and stained with silver, and bands were compared with a DNA fragment size marker (100-bp DNA ladder). The banding pattern was compared with the reference strains: *L*. *(V*.*) braziliensis* (MHOM/BR/1975/M2903), *L*. *(L*.*) amazonensis* (IFLA/BR/1967/PH8), *L*. *(V*.*) guyanensis* (MHOM/BR/1975/M4147), *L*. *(V*.*) shawi* (MCEB/BR/1984/M8408), *L*. *(V*.*) lainsoni* (MHOM/BR/1981/M6426), and *L*. *(V*.*) naiffi* (MDAS/BR/1979/M5533).

#### Sequencing

The ITS1-rDNA was amplified by conventional PCR using the primers L5.8S: 5′-TGATACCACTTATCGCACTT-3′ and LITSR: 5′-CTGGATCATTTTCCGATG-3′ [14]. Amplification reactions were performed in volumes of 50 μL. Amplicons from the PCR-positive samples (300–350 bp, depending on the species) were visualized on a 2% agarose gel and purified using the Wizard SV Gel kit and PCR Clean-up System kit (Promega, Madison, USA). The products were then sequenced with the same primers used in the PCR assay. Sequencing was performed on an automated sequencer at Plataforma de Sequenciamento Genômico ABI-3730 (Oswaldo Cruz Institute/FIOCRUZ).

Sequence alignment was performed using SeqMan Pro (DNASTAR) and comparisons were conducted with *Leishmania* reference strains sequences obtained from the GenBank database. Phylogenetics analyses with the evolutionary history was inferred using the maximum likelihood method based on the Jukes–Cantor model, and the sequences were aligned using Molecular Evolutionary Genetic Analysis (MEGA) version 6. This same software was used to calculate a distance matrix and the genetic distance percentage between the test samples and reference strains of *Leishmania* spp.

### Geospatial analysis

Georeferencing of each residence address and its respective LLI was performed using the online cartographic platform Google Earth, OpenStreetMap, or Bing Maps [30], with the geodetic reference system WGS 84 (World Geodetic System 1984). For LLI, georeferencing was performed from the centroid of the municipality, state, or country. Georeferencing was not possible in cases with two or more LLI, which were classified as undetermined LLI.

The maps were developed to represent the spatial distribution of *Leishmania* species according to the residence address of patients, in RJ state and RJ municipality. In the analysis of flows, maps were generated with the direction of the flows from the place of origin (LLI) to the destination (PR), also indicating the density (number of cases) and *Leishmania* species. The maps were constructed using ArcGIS 9.1 (Environmental Systems Research Institute, Redlands, CA, USA), using cartographic bases of the world, Brazil, RJ state, and RJ municipality obtained from the Brazilian Institute of Geography and Statistics (IBGE).

### Statistical analysis

Data entry was carried out through Microsoft Excel software and analyzed using SPSS software version 16 (SPSS Inc., Chicago, IL, USA). The frequencies (%) of categorical variables (sex, clinical form, country of infection, Brazilian region of infection, *Leishmania* species identified, and reason for exposure) were described, as well as the median (minimum–maximum) of the continuous variable age, as this was determined to be nonparametric using the Shapiro–Wilk normality test. Pearson´s chi-squared or Fisher’s exact test were used to verify the association between categorical variables. The Mann–Whitney U test was used to compare the median of age according to reason for exposure. A *p-*value<0.05 was considered statistically significant.

### Ethics statement

This study was approved by the Ethics Committee of NIID-FIOCRUZ (license number 1.591.506), all medical data analyzed were anonymized and all patients signed an informed consent form.

## Results

Of the 900 patients diagnosed with ATL at NIID-FIOCRUZ from 2000 to 2015, 195 (21.7%) patients with permanent or temporary residence in RJ state reported the LLI as other countries, other Brazilian states, or other regions of RJ state but different to their residence address. Of these patients, 24 were excluded because they had undetermined LLI and no *Leishmania* species identified. The final sample included 171 patients: 81 migrants (47.4%), 48 business travelers (28.1%), 33 leisure travelers (19.3%) and 9 undetermined (5.2%).

Among patients who were business travelers, the professions reported were military personnel (n = 30, 62.5%), electrician, oil or geology technician (n = 3, 6.2%), agronomist or forestry engineer (n = 2, 4.2%), and other professions with one patient each (n = 13, 27.1%). Clinical epidemiological data from the three groups of patients with determinate LLI are shown in Table 1.

**Table 1:** Sociodemographic and clinical characteristics of 162 patients* diagnosed with American tegumentary leishmaniasis at NIID-FIOCRUZ from 2000 to 2015.

	Migrants	Business Travelers	Leisure Travelers	TOTAL
	n = 81 (50%)	n = 48 (29.6%)	n = 33 (20.4%)	n = 162 * (100%)
**Sex** ^**a1**^^,^ ^**a2**^				
Female	22 (27.16%)	5 (10.42%)	14 (42.42%)	**41 (25.31%)**
Male	59 (72.84%)	43 (89.58%)	19 (57.58%)	**121 (74.69%)**
**Total**	81 (100.00%)	48 (100.00%)	33 (100.00%)	**162 (100.00%)**
**Age** ^**b1**^^,^ ^**b2**^				
Median (minimum–maximum)	44 (min 4—max 83)	30 (min 20 –max 56)	29 (min 12 –max 75)	**-**
**Region of Origin**				
**Brazil**				
North ^**c1**^^,^ ^**c2**^	5 (6.17%)	33 (68.75%)	4 (12.12%)	**42 (25.93%)**
Northeast ^**d1**^^,^ ^**d2**^^,^ ^**d3**^	40 (49.38%)	2 (4.17%)	7 (21.21%)	**49 (30.25%)**
South	-	-	1 (3.03%)	**1 (0.62%)**
Southeast ^**e1**^^,^ ^**e2**^	31 (38.27%)	4 (8.33%)	17 (51.52%)	**52 (32.10%)**
Midwest	1 (1.23%)	3 (6.25%)	2 (6.06%)	**6 (3.70%)**
Indeterminate	1 (1.23%)	4 (8.33%)	1 (3.03%)	**6 (3.70%)**
**Other countries**				
Bolivia	1 (1.23%)	1 (2.08%)	-	**2 (1.23%)**
Ecuador	-	1 (2.08%)	-	**1 (0.62%)**
French Guiana	2 (2.47%)	-	-	**2 (1.23%)**
Israel	-	-	1 (3.03%)	**1 (0.62%)**
**Total**	81 (100.00%)	48 (100.00%)	33 (100.00%)	**162 (100.00%)**
**Clinical forms** ^**f1**^^,^ ^**f2**^				
Cutaneous	43 (53.09%)	45 (93.75%)	26 (78.79%)	**114 (70.37%)**
Mucosal and Mucocutaneous**	38 (46.91%)	3 (6.25%)	7 (21.21%)	**48 (29.63%)**
**Total**	81 (100.00%)	48 (100.00%)	33 (100.00%)	**162 (100.00%)**

* The 162 patients were residents in Rio de Janeiro state and with a determined likely location of infection. Nine patients with more than one reason for exposure, classified as undetermined, were removed from this analysis.

** For statistical calculation, cases of mucocutaneous leishmaniasis were included in the mucosal leishmaniasis group. p < 0.05 values are considered significant.

**ª**^**1**^ p = 0.024: Migrant x Business Travelers

**ª**^**2**^ p = 0.001: Business Travelers x Leisure Travelers

^**b1**^ p<0.001: Migrant x Business Travelers

^**b2**^ p = 0.028: Migrant x Leisure Travelers

^**c1**^ p<0.001: Migrant x Business Travelers

^**c2**^ p<0.001: Business Travelers x Leisure Travelers

^**d1**^ p<0.001: Migrant x Business Travelers

^**d2**^ p = 0.028: Business Travelers x Leisure Travelers

^d3^ p = 0.005: Migrant x Leisure Travelers

^**e1**^ p<0.001: Migrant x Business Travelers

^**e2**^ p<0.001: Business Travelers x Leisure Travelers

^**f1**^ p<0.001: Migrant x Business Travelers

^**f2**^ p<0.011: Migrant x Leisure Travelers

Of the 171 patients, 117 (68.4%) were diagnosed via isolation of *Leishmania* spp. in culture. The diagnosis was confirmed in the remaining patients using at least one of the following criteria: visualization of amastigotes forms of *Leishmania* on histopathological examination or direct examination, demonstration of parasitic kDNA by PCR, clinical epidemiological criteria associated with serological test positivity (ELISA) or Montenegro skin test.

### *Leishmania* species typing

It was not possible to recover any parasites from 13 samples due to contamination by bacteria or fungi or no growth in culture after cryopreservation. Of the 117 parasites isolated in culture, 104 (88.9%) were characterized as belonging to one of four different *Leishmania* species and one genetic variant (Tables 2 and 3). Eleven (29.7%) of the business travelers were infected by species other than *L*. *(V*.*) braziliensis* or its variant, compared with four (9.1%) of the migrants (p = 0.017).

**Table 2:** Identification of 104 *Leishmania* species regarding the reason for exposure.

Species Typing	Migrants	Business Travelers	Leisure Travelers	Undetermined reason for exposure	TOTAL
***L*. *(V*.*) braziliensis***	40	22	13	9	**84 (80.8%)**
***L*. *(V*.*) braziliensis* (variant)**	-	4	-	-	**4 (3.8%)**
***L*. *(V*.*) naiffi***	-	8	-	-	**8 (7.7%)**
***L*. *(V*.*) guyanensis***	3	3	1	-	**7 (6.7%)**
***L*. *(L*.*) amazonensis***	1	-	-	-	**1 (1%)**
**TOTAL**	**44 (42.3%)**	**37 (35.6%)**	**14 (13.5%)**	**9 (8.6%)**	**n = 104 (100%)**

**Table 3:** Identification techniques used for typing of 104 *Leishmania* species isolated from travelers and migrants who were patients attended at NIID-FIOCRUZ from 2000 to 2015.

	A Isoenzymes (MLEE)	B PCR–RFLP	A and B	A and B and Sequencing	TOTAL	
***L*. *(V*.*) braziliensis***	68	16	-	-	**84 (80.8%)**	**88 (84.6%)**
***L*. *(V*.*) braziliensis* (variant)**	1	-	2	1	**4 (3.8%)**	
***L*. *(V*.*) naiffi***	1	3	4	-	**8 (7.7%)**	
***L*. *(V*.*) guyanensis***	6	1	-	-	**7 (6.7%)**	
***L*. *(L*.*) amazonensis***	1	-	-	-	**1 (1%)**	
**TOTAL**	**77 (74%)**	**20 (19.2%)**	**6 (5.8%)**	**1 (1%)**	**104 (100%)**	

NIID = Evandro Chagas National Institute of Infectious Diseases; MLEE = Multilocus enzyme electrophoresis

### Genetic variants of *L*. *(V*.*) braziliensis*

In the analysis of seven enzymatic loci using the MLEE technique, the four samples (P1, P2, P3 and P4) showed a profile of *L*. *(V*.*) braziliensis* in two, three, four, or five enzymatic loci and variant profiles in the other loci (Supplementary data–S1 Table).

**Fig 1:**
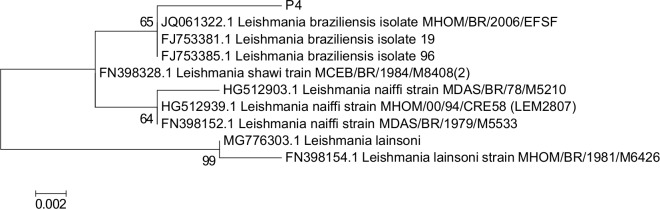
Phylogenetic analysis demonstrating *Leishmania (Viannia)* species with sequences of ITS1-rDNA. The variant *Leishmania braziliensis* P4 sequence (GenBank accession number MN508061) is present, with 0.8% of genetic distance when compared to other sequences of *Leishmania braziliensis*. The evolutionary history was inferred using maximum likelihood based on the Jukes–Cantor model; sequences were aligned using MEGA version 6 software. Outgroup: *Leishmania lainsoni*. Bootstrap test (1000 replications) are shown next to the branches, and GenBank accession numbers are in front of the species names.

The isolate P4, with an LLI in Acre state, presented a profile of *L*. *(V*.*) braziliensis* in two loci and a variant profile in five enzymatic loci. According to PCR-RFLP analysis, P4 presented an *L*. *(V*.*) braziliensis* / *L*. *(V*.*) naiffi* profile after digestion with *Hae*III enzyme and a *L*. *(V*.*) shawi* profile after digestion with *Bst*UI enzyme. To confirm the species, genetic sequencing of the ITS1-rDNA was performed and the consensus sequence is shown as supplementary data–S1 Consensus_Sequence. Phylogenetic analyses (Fig 1) and genetic distance percentage between the P4 isolate and other *Leishmania* species (Table 4) confirmed the P4 isolate to be a genetic variant of *L*. *(V*.*) braziliensis*.

**Table 4:** Genetic distance percentage between P4 isolate and other *Leishmania* species in ITS1-rDNA sequences obtained from GenBank.

*Leishmania* species (GenBank code)	Genetic Distances (%) for P4 isolate *
*L*. *braziliensis* (JQ061322/FJ753385/FJ7533/81)	0.8%
*L*. *shawi* (FN398328)	1.2%
*L*. *naiffi* (HG512939/FN398152)	1.6%
*L*. *naiffi* (HG512903)	2.1%
*L*. *lainsoni* (MG776303)	3.3%
*L*. *lainsoni* (FN398154)	3.8%

*GeneBank code MN508061

Of the 104 *Leishmania* species identified, 101 were from Brazil and 3 from other countries. In the South, Southeast and Midwest Regions 100% of the species identified were *L*. *(V*.*) braziliensis*. All the four *L*. *(V*.*) braziliensis* (variants) identified were from the North Region. In the North region, of the 26 *Leishmania* species identified, 11 (42.3%) were different from *L*. *(V*.*) braziliensis* and in the Northeast region, of the 26 *Leishmania* species identified, 3 (11.5%) were different of *L*. *(V*.*) braziliensis* (p = 0,012) (Tables 5 and Supplementary data–S2 Table). The predominant clinical form was CL (n = 80; 77%), followed by ML (n = 17, 16.3%) and MCL (n = 7; 6.7%) (Supplementary data–S2 Table).

**Table 5:** Species of *Leishmania* identified according to likely location of infection in Brazilian regions or other countries.

	Brazil						Other Countries	TOTAL
	North Region	Northeast Region	Southeast Region	Midwest Region	South Region	Indeterminate Region		
***L*. *(V*.*) braziliensis***	11	23	39	2	1	6	2	**84 (80.8%)**
***L*. *(V*.*) braziliensis* (variant)**	4	-	-	-	-	-	-	**4 (3.8%)**
***L*. *(V*.*) naiffi***	7	-	-	-	-	1	-	**8 (7.7%)**
***L*. *(V*.*) guyanensis***	4	2	-	-	-	-	1	**7 (6.7%)**
***L*. *(L*.*) amazonensis***	-	1	-	-	-	-	-	**1 (1%)**
**TOTAL**	**26 (25%)**	**26 (25%)**	**39 (37.5%)**	**2 (1.9%)**	**1 (1%)**	**7 (6.7%)**	**3 (2.9%)**	**n = 104 (100%)**

### Distribution of *Leishmania* species according to place of residence (PR)

**Fig 2:**
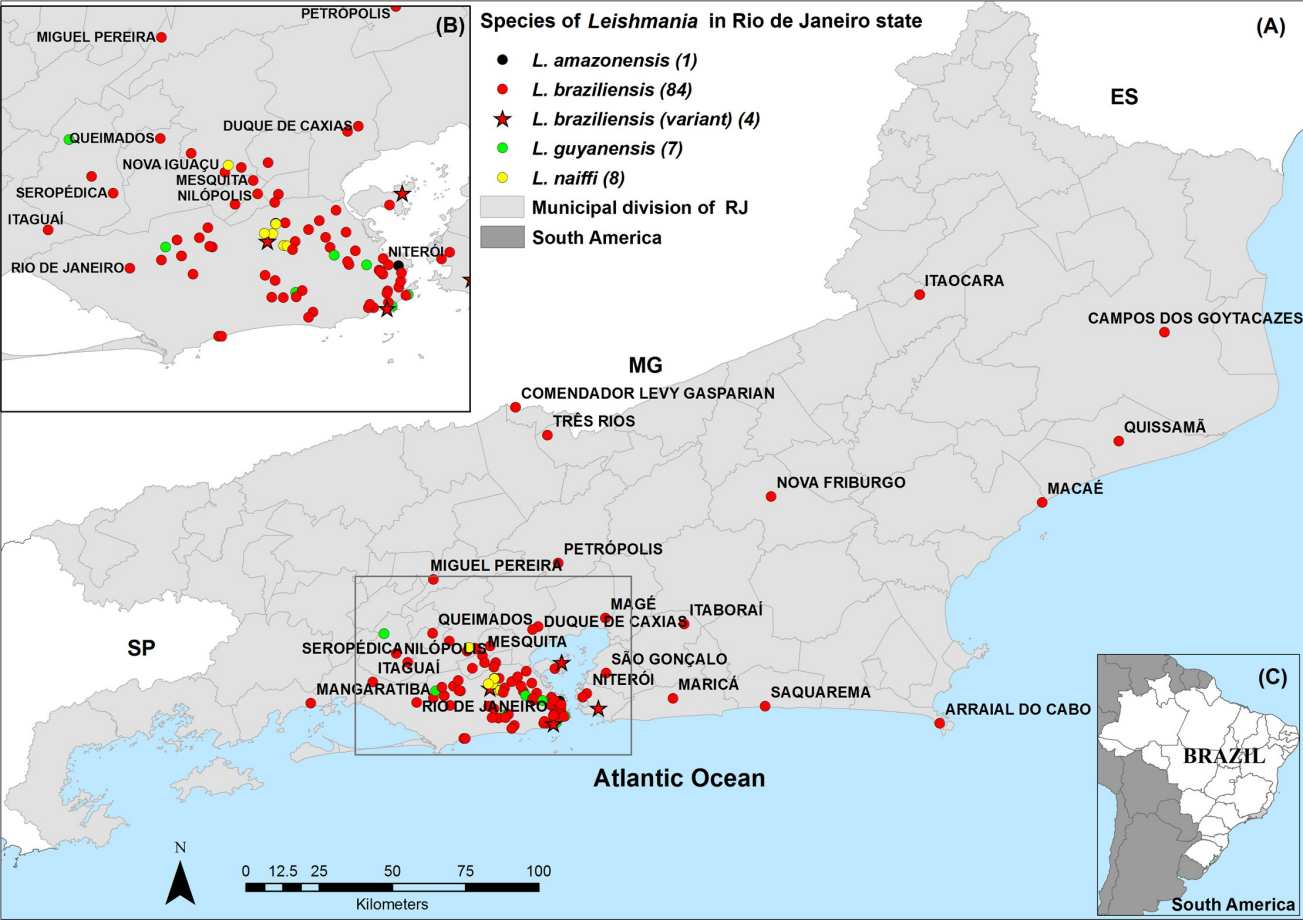
Species of *Leishmania* in Rio de Janeiro (RJ) state. The 104 typed species of *Leishmania* were plotted on the map by municipalities of RJ state (A); The municipality of Rio de Janeiro and some neighboring municipalities are highlighted to facilitate the visualization of the identified *Leishmania* species (B); Location of RJ state on maps of Brazil and South America (C). SP = São Paulo state; MG = Minas Gerais state; ES = Espírito Santo state. This map was created using ArcGIS 9.1 software and cartographic bases obtained from the Brazilian Institute of Geography and Statistics (https://ibge.gov.br/).

We determined the geographic distribution of 104 *Leishmania* species identified, according to the residence address of patients (Fig 2).

**Fig 3:**
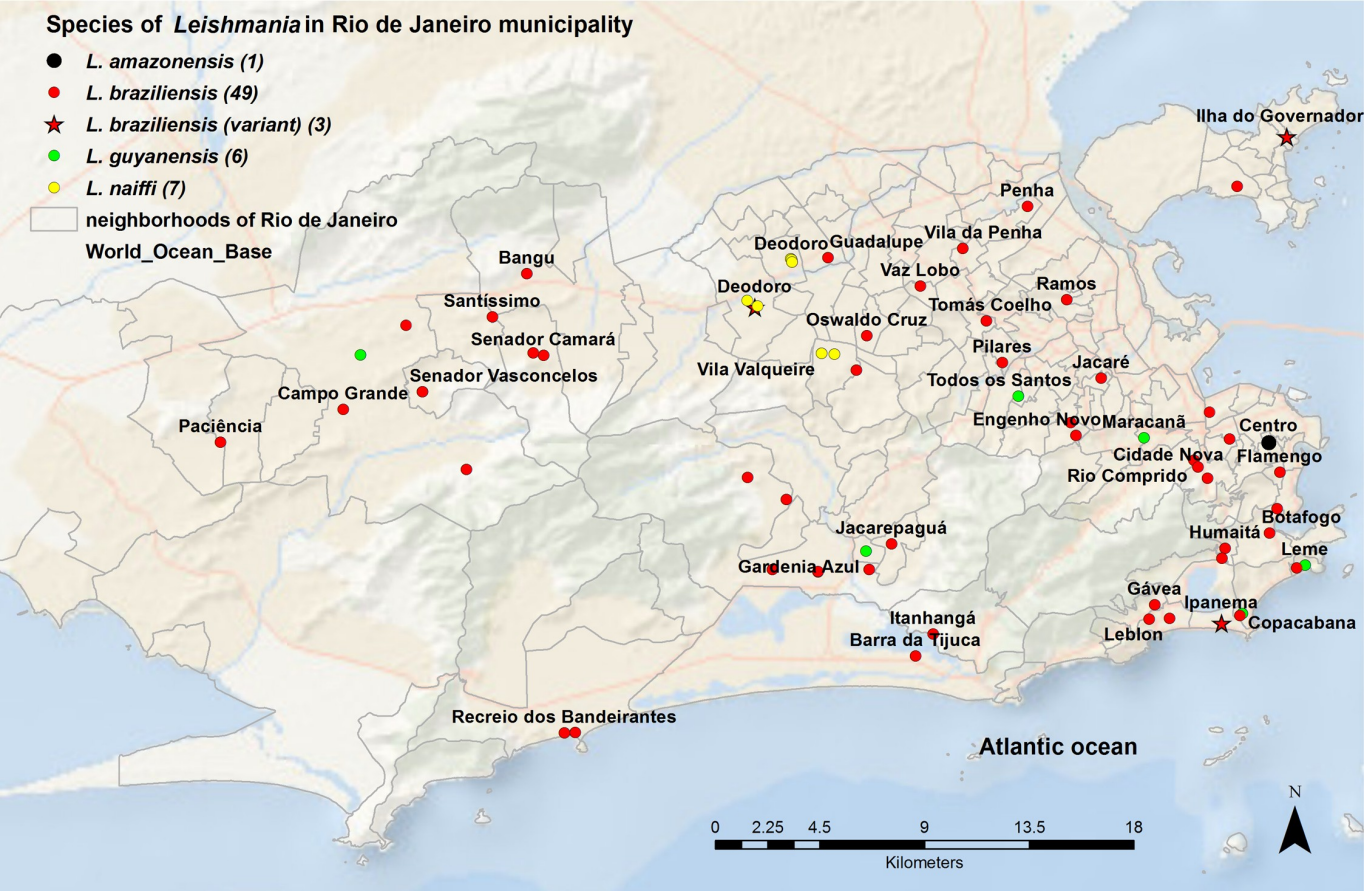
Species of *Leishmania* in Rio de Janeiro (RJ) municipality. The 66 species of *Leishmania* identified were plotted on the map by neighborhoods of RJ municipality, according to the residence address of the patients in RJ municipality. This map was created using ArcGIS 9.1 software and cartographic bases obtained from the Brazilian Institute of Geography and Statistics (https://ibge.gov.br/).

The largest number of species identified (n = 66) and the highest diversity was in the municipality of RJ: *L*. *(V*.*) braziliensis* (n = 49), *L*. *(V*.*) braziliensis* variant (n = 3), *L*. *(V*.*) naiffi* (n = 7), *L*. *(V*.*) guyanensis* (n = 6), and *L*. *(L*.*) amazonensis* (n = 1) (Fig 3).

**Fig 4:**
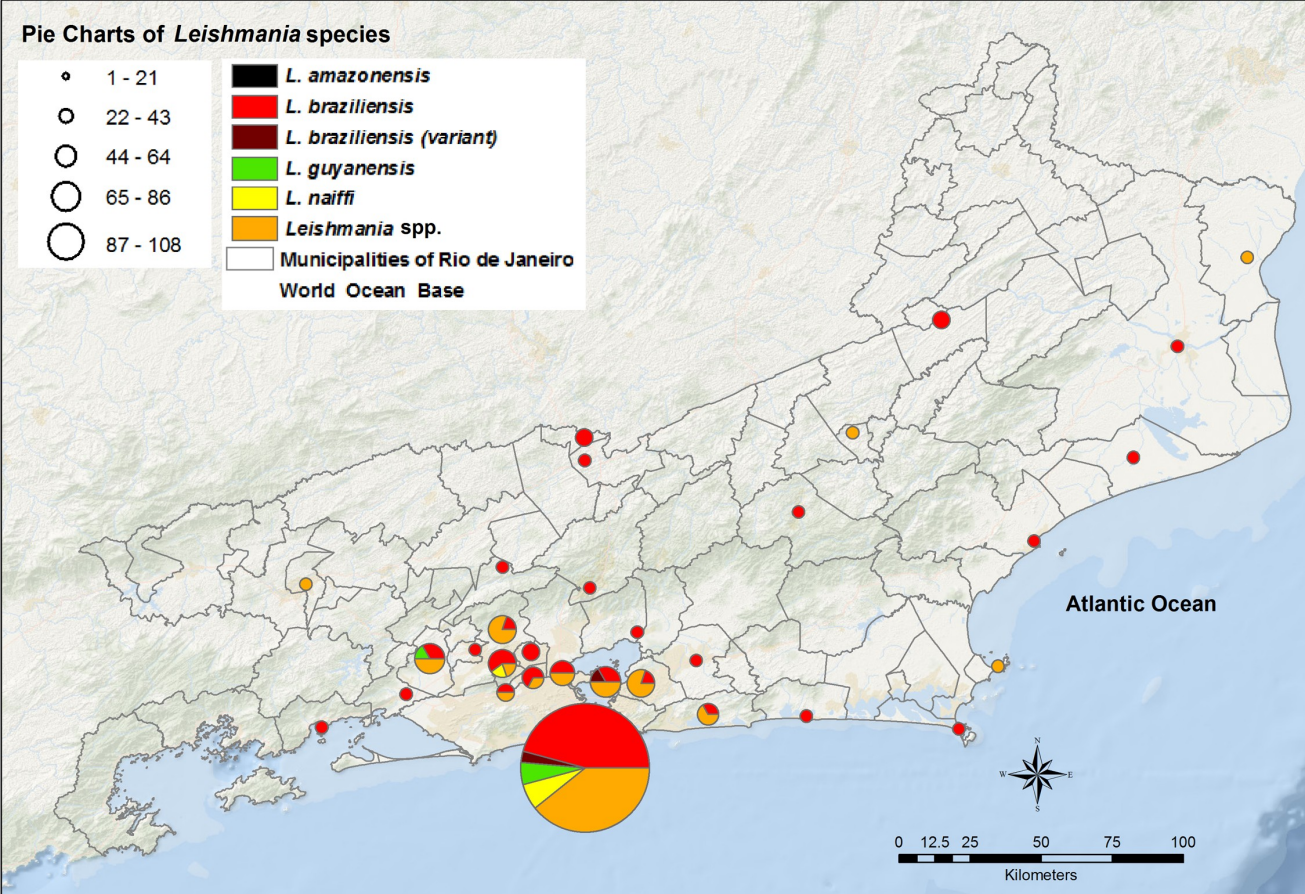
Pie charts of *Leishmania* species, representing the aggregated distribution of *Leishmania* species by municipalities of Rio de Janeiro state. The size of pie chart is proportional to the amount of samples of *Leishmania* in each municipality. Uncharacterized species were represented by an orange color (*Leishmania* spp.). This map was created using ArcGIS 9.1 software and cartographic bases obtained from the Brazilian Institute of Geography and Statistics (https://ibge.gov.br/).

In the other municipalities of RJ state, 38 species were identified: *L*. *(V*.*) braziliensis* (n = 35), *L*. *(V*.*) braziliensis* variant (n = 1), *L*. *(V*.*) naiffi* (n = 1), and *L*. *(V*.*) guyanensis* (n = 1). In Niteroi municipality, *L*. *(V*.*) braziliensis* (n = 2) and *L*. *(V*.*) braziliensis* (variant) (n = 1) were detected. In Nova Iguaçu municipality, *L*. *(V*.*) braziliensis* (n = 3) and *L*. *(V*.*) naiffi* (n = 1) were identified. *L*. *(V*.*) braziliensis* (n = 2) and *L*. *(V*.*) guyanensis* (n = 1) were detected in Seropedica municipality. In the other 23 municipalities, *L*. *(V*.*) braziliensis* as a single species was identified (Figs 2 and 4).

### Flow maps

**Fig 5:**
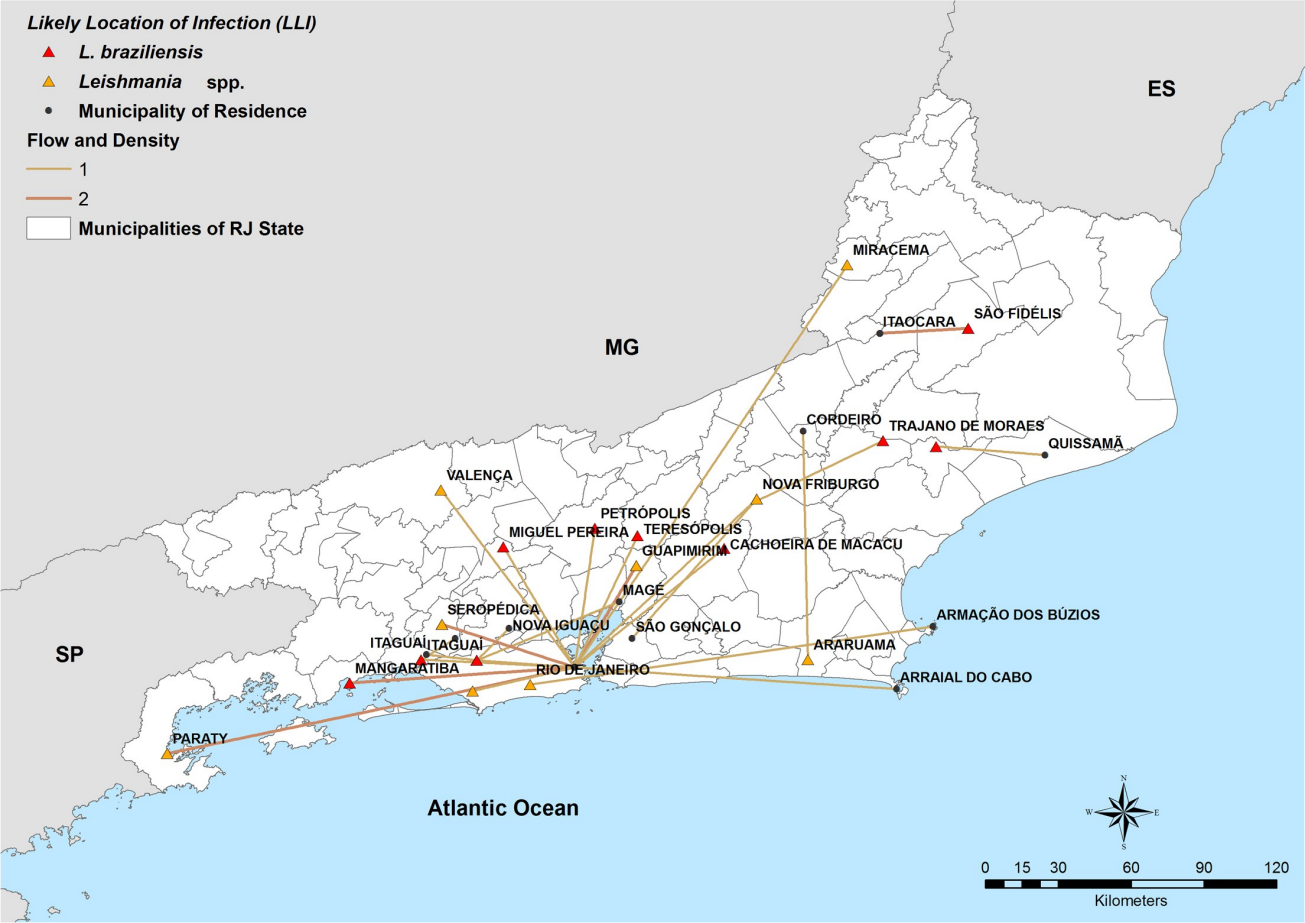
Flow map with density of cases in Rio de Janeiro state. Municipalities of residence are represented by circles and likely location of infection by triangles. Unidentified species (*Leishmania* spp.) are represented by an orange triangle and species identified as *L*. *(V*.*) braziliensis* by a red triangle. Yellow lines represent the migratory flow of one case (density 1) and brown lines represent the migratory flow of two cases (density 2). This map was created using ArcGIS 9.1 software and cartographic bases obtained from the Brazilian Institute of Geography and Statistics (https://ibge.gov.br/).

The flow map of RJ state (n = 30), considering the municipality of residence in RJ state and its respective likely location of infection within RJ state is shown in Fig 5.

**Fig 6:**
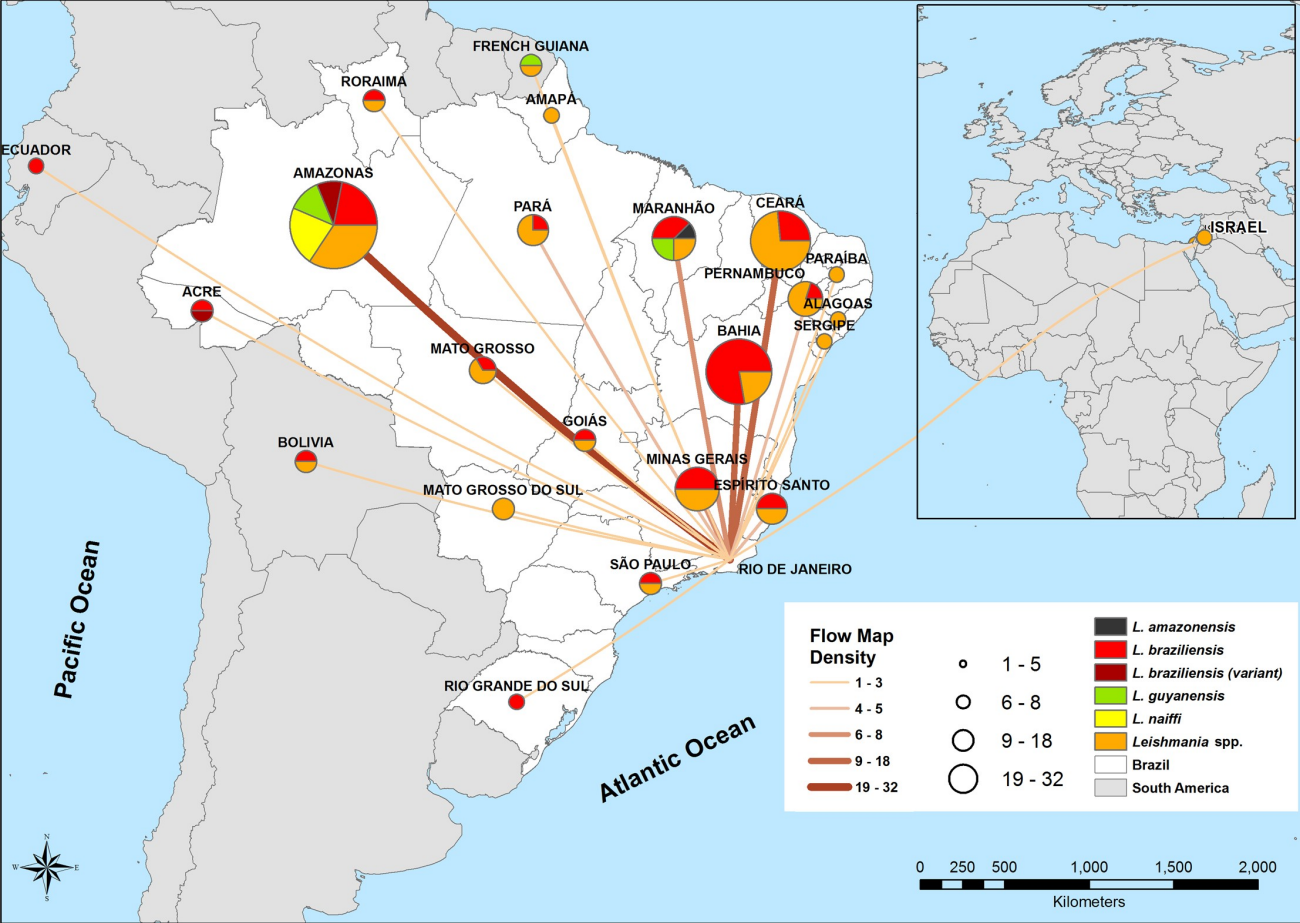
Map of flows to Rio de Janeiro state, with density of cases from other countries and Brazilian states. The density of cases was divided into five categories, differentiated by color and thickness. The lines are varied in width to represent the number of cases, with a broader line indicating greater flow. The width of the line is proportional to the flow. Pie charts represent the aggregated distribution of *Leishmania* species by country and state. This map was created using ArcGIS 9.1 software and cartographic bases obtained from the Brazilian Institute of Geography and Statistics (https://ibge.gov.br/).

Fig 6 illustrates the flow map, including Brazilian states (n = 112) and countries (n = 6) with *Leishmania* species aggregated according to LLI and density of cases. Uncharacterized species were identified as *Leishmania* spp. Of the six cases from other countries, three were identified: *L*. *(V*.*) guyanensis* from French Guiana, *L*. *(V*.*) braziliensis* from Bolivia, and *L*. *(V*.*) braziliensis* from Ecuador. It was not possible to identify the other three species from Bolivia, French Guiana, and Israel as they had negative (n = 1) or no culture results (n = 2).

The highest densities were among patients with LLI in the following states: Amazonas (n = 32), Bahia (n = 18), Ceará (n = 15), Minas Gerais (n = 8), and Maranhão (n = 8). The most diverse flows of *Leishmania* species came from the Amazonas state with the following species: *L*. *(V*.*) braziliensis*, *L*. *(V*.*) braziliensis* variant, *L*. *(V*.*) naiffi*, and *L*. *(V*.*) guyanensis*, as well as from the Maranhão state: *L*. *(V*.*) braziliensis*, *L*. *(V*.*) guyanensis*, and *L*. *(L*.*) amazonensis* (Fig 6).

## Discussion

Among the countries of South America, Brazil is the country that receives the highest number of international tourists. RJ state receives millions of domestic and international tourists annually, as it is the main leisure destination in Brazil [31]. Ecotourism, with outdoor adventure and leisure activities that take place in endemic areas, increases the risk of contracting ATL, which is among the 10 most common dermatological diseases occurring among tourists returning from tropical countries [32]. In this study, ATL cases were detected among both leisure travelers and professionals traveling to work in endemic regions, such as biologists, forest engineers, police officers, technicians, and military personnel. Among these professionals, the highest number of cases was detected among military personnel who reported training in the Amazonian forest. These data reinforce the emergence of leishmaniasis among travelers and the importance of diagnostic, prevention, and control measures for professionals and tourists traveling to endemic regions [27,28]. In addition to business and leisure travelers, migrants also facilitate the flow of different *Leishmania* species to RJ state.

It is important highlight that the samples analyzed represent the casuistry of a reference center, responsible for the care and diagnosis of 25% of the ATL cases reported in RJ state, including about 75% of the more serious cases. It was also not possible to identify the *Leishmania* species of some patients because they had no parasitic isolation in culture or had their samples lost. Furthermore, other limitations of the study were some patients reported two or more LLI or reason for exposure, both being classified as undetermined.

Analysis of the reasons for exposure showed significant differences in relation to the regions of origin. The reasons for exposure among patients in the North Region were more frequently associated with business travel, and those in the Northeast Region were associated with migration; in the Southeast Region, exposure was more associated with leisure travel, although this region also had a large number of migrants.

In this study, there was a greater number of ATL cases among young adults and men. Regarding sex, there were more cases among men than women, both in migrants and travelers. However, this sex difference was smaller among leisure travelers. Among working travelers, there was a higher number of cases of ATL among men. There was a significant difference with respect to age between migrants and travelers. The median age was higher among migrants than among business and leisure travelers, demonstrating that travelers in the study area are younger. The predominant clinical form found was CL; ML and MCL were more common among migrants than business and leisure travelers. Although our study population consisted of migrants and travelers, these data are in agreement with reported cases in RJ between 2007 and 2017, with a greater number of ATL cases in adults, men, and those with CL as the main clinical form [33]. However, although CL was the predominant form, the number of ML and MCL cases was higher than that reported in RJ state and in Brazil. This can be explained by the fact that this study was carried out at a reference center, with greater availability of specific tests and doctors specialized in otolaryngology and dermatology, thus enhancing the ability to diagnose ML and MCL [29].

The main diagnostic technique was parasite isolation in culture of cutaneous or mucosal lesion fragments. Despite the limitations already described, use of MLEE allowed us to identify most species, corroborating what has been described in the literature in which this technique remains the gold standard for typing parasites of the *Leishmania* genus [7]. However, in cases where etiological identification with MLEE was not possible, a PCR-RFLP assay for the heat-shock protein 70 gene (*hsp70*C) proved to be a good alternative for discrimination of *Leishmania* species, as described in other studies [11,34]. In only one case, it was necessary to use DNA Sanger sequencing and the target used (ITS1-rDNA) proved effective for etiological identification. Another advantage is that this technique can be applied directly to clinical samples, without the need of parasitic isolation in culture [13,14].

In the New World, parasites of the subgenus *Viannia*, the main agents of ATL, represent a group of microorganisms with considerable intraspecific variability, which may hamper taxonomic classification and epidemiological studies [35,36]. The main species identified in this study was *L*. *(V*.*) braziliensis*, corroborating that this is the most frequent species in Brazil [2]. In the North and Northeast regions of Brazil, other species have also been identified, such as *L*. *(V*.*) naiffi*, *L*. *(V*.*) guyanensis*, *L*. *(L*.*) amazonensis*, and genetic variants of *L*. *(V*.*) braziliensis*. The only species from the South, Southeast, and Midwest regions was *L*. *(V*.*) braziliensis*, with no variants or other species identified. It is important emphasize that some species of sandflies vectors of the *Leishmania* species identified in this study have already been described in RJ state: *Lu*. *(N*.*) flaviscutellata*, vector of *L*. *(L*.*) amazonensis*, *Lu*. *(P*.*) paraensis* and *Lu*. *(P*.*) ayrozai*, vectors of *L*. *(V*.*) naiffi*. There is no report in the literature about the presence of vectors of *L*. *(V*.*) guyanensis* in RJ state [5].

The Brazilian states with the highest density of ATL cases were Amazonas, Ceará, and Bahia. However, states that presented the greatest diversity of species were Amazonas and Maranhão; the latter state is located in the Northeast Region of Brazil. *Leishmania (L*.*) amazonensis* was identified in a single case isolated from a migrant patient from Maranhão, who had CL.

In the present study, 35 species from Amazon region were identified and *L*. *(V*.*) braziliensis* was the most frequent. Four cases identified as genetic variants of *L*. *(V*.*) braziliensis* were from this region, three from Amazonas state and one from Acre state, both located in the Amazon forest biome. It was only possible to confirm the etiological identity of the sample from Acre using sequencing, as this isolate showed great variability in both the isoenzyme profiles and using PCR-RFLP. From an epidemiological point of view, the complex cycle of transmission in which individuals are exposed, basically in the Amazonian area, contributes to the genetic diversity of *Leishmania* parasites [37,38]. Eco-epidemiological interactions occurring in such endemic areas may be reflected in the dynamics of circulating populations of *L*. *(V*.*) braziliensis*, and finding genetic variants of this species may be a consequence. It is well known that heterogeneous subpopulations circulate among humans and vectors as well as domestic and sylvatic animals in simple and complex epidemiological situations [39]. In addition, the polyclonality of the initial inoculum has been previously reported [35] and genetic variability of *L*. *(V*.*) braziliensis* has also been identified among patients and among different lesions in the same patient [40]. Reinforcing these arguments, other authors have also demonstrated an association between the diversity of vectors involved in the transmission of *L*. *(V*.*) braziliensis* and the genetic diversity of these parasites [41]. In order to complement the data of this study, future studies with more discriminatory methods like Multilocus Sequence Typing (MLST), Multilocus Microsatellite Typing (MLMT) and Restriction-site associated DNA sequencing (RAD-seq) could be performed to evaluate the intra and inter-specific genetic diversity of these isolates of *Leishmania* spp [42,43].

In the Amazon region, there are at least seven circulating species of dermotropic *Leishmania*, with *L*. *(V*.*) guyanensis* being the most frequent species in this region [44]. However, in this study, 35 species from the Amazon region were identified and *L*. *(V*.*) braziliensis* was the most prevalent. *L*. *(V*.*) guyanensis* and *L*. *(V*.*) naiffi* were found in the same number of cases. Our results are in agreement with those of previous studies, which suggest that *L*. *(V*.*) naiffi* may be more frequent in the Amazon region than is currently recognized [45,46]. Another case of infection by *L*. *(V*.*) naiffi* was identified, but the patient had an undetermined LLI as he reported a trip to the Amazon and also to the Pantanal biome, in Mato Grosso state. It is important to mention that all identified cases of *L*. *(V*.*) naiffi* were among military personnel who were training in the Amazonian forest.

Two cases from French Guiana were identified, one as a case of CL in which the species could not be identified, and the other as a case of MCL caused by *L*. *(V*.*) guyanensis*, the most frequent species in that country [47].

Two cases were from Bolivia; one was a case of ML in Santa Cruz de la Sierra, the largest and most populous city in the country, and the species was identified as *L*. *(V*.*) braziliensis*, the most frequent species in that country [48]. The other case was CL, in which the species could not be identified.

There was one case of CL from Ecuador, in which the species was identified as *L*. *(V*.*) braziliensis*. This species is well distributed throughout the Ecuadorian Amazon region and has also been observed on the Pacific Coast. Currently, eight dermotropic species of *Leishmania* are described in Ecuador: *Leishmania (L*.*) mexicana*, *L*. *(L*.*) amazonensis*, *L*. *(L*.*) major-like*, *L*. *(V*.*) panamensis*, *L*. *(V*.*) braziliensis*, *L*. *(V*.*) naiffi*, *L*. *(V*.*) lainsoni*, and *L*. *(V*.*) guyanensis*, the latter of which is the most frequent species in that country [49].

There was also one case from Israel, with no parasitic isolation in culture; therefore, no species of *Leishmania* could be identified. Leishmaniasis is endemic in Israel and is increasing in frequency. The pathogens that cause CL in Israel are *Leishmania major* and *Leishmania tropica* [50].

The diversity of clinical manifestations of ATL is related to the diversity of *Leishmania* species and also to the host immune response. Thus, clinical and therapeutic management may be different depending on the species of *Leishmania* involved. The treatment with meglumine antimoniate has already been shown to be effective in patients from different Brazilian regions, except in areas where *L*. *(V*.*) guyanensis* infections are common, which has poor response to antimonials and should be initially treated with pentamidine [51,52].

The transmission dynamics of ATL in the state of RJ has been variable, with a decreasing trend in the number of cases reported in recent years [33]. For a better understanding of the epidemiology of leishmaniasis, it is important to know the circulating *Leishmania* species in a determined geographical area. This work is the first contribution to the knowledge of the routes of *Leishmania* species introduced in RJ state by migrants and travelers patients. In addition to *L*. *(V*.*) braziliensis*, which was the most common species, other species have also been identified, such as *L*. *(V*.*) guyanensis*, *L*. *(V*.*) naiffi*, *L*. *(L*.*) amazonensis*, and genetic variants of *L*. *(V*.*) braziliensis*. It is important to emphasize that ATL is a vector-borne disease and only the presence of these migrants and travelers patients in RJ state does not guarantee the autochthonous transmission, being necessary the adaptation of these different species of *Leishmania* to environmental conditions as well as the presence of reservoirs and phlebotomine vectors in this region. The question remains whether the establishment and autochthonous transmission of these imported species is possible. This can only be answered using knowledge of the geographic distribution of these *Leishmania* species as a basis for future studies on typing of *Leishmania* species isolated from patients diagnosed in the state of RJ, and associations of the presence of these species with the distribution of hosts, vectors, climatic data, vegetation, and socioenvironmental conditions. Such research will contribute with important elements needed for the surveillance and control of leishmaniasis in RJ state.

List of accession numbers/ID numbers for genes and proteins mentioned in the text: Name/Gene ID LMJF_28_2780: heat-shock protein (*hsp*)*70* gene on chromosome 28 of *Leishmania major* [strain Friedlin NC_007269.2 (1060574.1062550, complement) accession FR796424 CT005266] Font: https://www.ncbi.nlm.nih.gov/gene. ID number EC 1.1.1.43: 6-phosphogluconate dehydrogenase (6PGDH); ID number EC 5.3.1.9: glucose phosphate isomerase (GPI); ID number EC 3.2.2.1: nucleotidase (NH); ID number EC 1.1.1.49: glucose-6-phosphate dehydrogenase (G6PDH); ID number EC 5.4.2.2: phosphoglucomutase (PGM); ID number EC 1.1.1.40: malic enzyme (ME) and ID number EC 1.1.1.37 malate dehydrogenase (MDH). Font: https://www.genome.jp/kegg/

## Supporting information

S1 Checklist STROBE statement-checklist. (DOC)

S1 Table Enzymatic_Variants_loci_*L_braziliensis*. (DOCX)

S2 Table Likely_Location_of_Infection_Species_typing_and_associated_clinical_forms. (DOCX)

S1 Consensus Sequence_ITS-1_P4. (DOCX)
