# Supplementary material for: Geospatial analysis of tegumentary leishmaniasis in Rio de Janeiro state, Brazil from 2000 to 2015: Species typing and flow of travelers and migrants with leishmaniasis
Source: PLoS Negl Trop Dis. 2019 Nov 15;13(11):e0007748. doi: 10.1371/journal.pntd.0007748 (PMC6857848; doi:10.1371/journal.pntd.0007748)
Supplement: S1 Table — (DOCX) [file pntd.0007748.s002.docx]

**S1: Table. Identification of enzyme loci revealed by MLEE of samples classified as genetic variants of *Leishmania (Viannia) braziliensis*.**

| **Samples identified as genetic variants of *L. (V.) braziliensis* (n=4)** | | |
| --- | --- | --- |
|  | **Origin** | **Enzymatic Variants Loci by MLEE** |
| P1 | Amazonas | NH e ME |
| P2 | Amazonas | ME, GPI e 6PGDH |
| P3 | Amazonas | PGM, GPI, ME e MDH |
| P4 | Acre | NH, GPI, ME, PGM e MDH |

NH = nucleotidase; ME = malic enzyme; GPI = glucose phosphate isomerase; 6PGDH = 6-phosphogluconate dehydrogenase; PGM = phosphoglucomutase; MDH = malate dehydrogenase; G6PDH = glucose-6-phosphate dehydrogenase.
