# Supplementary material for: Geospatial analysis of tegumentary leishmaniasis in Rio de Janeiro state, Brazil from 2000 to 2015: Species typing and flow of travelers and migrants with leishmaniasis
Source: PLoS Negl Trop Dis. 2019 Nov 15;13(11):e0007748. doi: 10.1371/journal.pntd.0007748 (PMC6857848; doi:10.1371/journal.pntd.0007748)
Supplement: S2 Table — (DOCX) [file pntd.0007748.s003.docx]

**S3_Table: Identification of 104 *Leishmania* species, according to likely location of infection (country, region, and state) and the associated clinical forms.**

| **COUNTRY/REGION** | **LIKELY STATE OF INFECTION** | **SPECIES TYPING** | **CLINICAL FORMS** |
| --- | --- | --- | --- |
| BRAZIL  NORTH REGION (n=26) | ACRE (n=2) | *L. (V.) braziliensis* (n=1)  *L. (V.) braziliensis* (variant) (n=1) | CUTANEOUS (n=2) |
|  | AMAZONAS (n=21) | *L. (V.) naiffi* (n=7) | CUTANEOUS (n=7) |
|  |  | *L. (V.) braziliensis* (n=7) | CUTANEOUS (n=6)  MUCOCUTANEOUS (n=1) |
|  |  | *L. (V.) guyanensis* (n=4) | CUTANEOUS (n=4) |
|  |  | *L. (V.) braziliensis* (variant) (n=3) | CUTANEOUS |
|  | ACRE or AMAZONAS (n=1) | *L. (V.) braziliensis* (n=1) | CUTANEOUS |
|  | PARÁ (n=1) | *L. (V.) braziliensis* (n=1) | CUTANEOUS |
|  | RORAIMA (n=1) | *L. (V.) braziliensis* (n=1) | MUCOSAL |
| BRAZIL  NORTHEAST REGION  (n=26) | BAHIA (n=14) | *L. (V.) braziliensis* (n=14) | CUTANEOUS (n=11)  MUCOSAL (n=3) |
|  | CEARÁ (n=4) | *L. (V.) braziliensis* (n=4) | CUTANEOUS (n=4) |
|  | MARANHÃO (n=6) | *L. (V.) braziliensis* (n=3) | CUTANEOUS (n=1)  MUCOCUTANEOUS (n=1)  MUCOSAL (n=1) |
|  |  | *L. (V.) guyanensis* (n=2) | CUTANEOUS (n=2) |
|  |  | *L. (L.) amazonensis* (n=1) | CUTANEOUS |
|  | PERNAMBUCO (n=1) | *L. (V.) braziliensis* (n=1) | CUTANEOUS |
|  | ALAGOAS or CEARÁ (n=1) | *L. (V.) braziliensis* (n=1) | CUTANEOUS |
| BRAZIL  SOUTHEAST REGION  (n=39) | RIO DE JANEIRO (n=21) | *L. (V.) braziliensis* (n=21) | CUTANEOUS (n=16)  MUCOCUTANEOUS (n=3)  MUCOSAL (n=2) |
|  | MINAS GERAIS (n=4) | *L. (V.) braziliensis* (n=4) | CUTANEOUS (n=2)  MUCOSAL (n=2) |
|  | ESPÍRITO SANTO (n=2) | *L. (V.) braziliensis* (n=2) | CUTANEOUS (n=1)  MUCOSAL (n=1) |
|  | SÃO PAULO (n=1) | *L. (V.) braziliensis* (n=1) | MUCOSAL |
|  | RIO DE JANEIRO or ESPÍRITO SANTO (n=1) | *L. (V.) braziliensis* (n=1) | MUCOSAL |
|  | RIO DE JANEIRO or MINAS GERAIS (n=7) | *L. (V.) braziliensis* (n=7) | CUTANEOUS (n=5)  MUCOCUTANEOUS (n=1)  MUCOSAL (n=1) |
|  | RIO DE JANEIRO or ESPÍRITO SANTO or MINAS GERAIS (n=1) | *L. (V.) braziliensis* (n=1) | CUTANEOUS |
|  | RIO DE JANEIRO or MINAS GERAIS or SÃO PAULO (n=2) | *L. (V.) braziliensis* (n=2) | CUTANEOUS (n=1)  MUCOSAL (n=1) |
| BRAZIL  MIDWEST REGION  (n=2) | GOIÁS (n=1) | *L. (V.) braziliensis* (n=1) | CUTANEOUS |
|  | MATO GROSSO (n=1) | *L. (V.) braziliensis* (n=1) | CUTANEOUS |
| BRAZIL  SOUTH REGION  (n=1) | RIO GRANDE DO SUL (n=1) | *L. (V.) braziliensis* (n=1) | CUTANEOUS |
| BRAZIL INDETERMINATE REGION (n=7) | DISTRITO FEDERAL or BAHIA or GOIÁS (n=1) | *L. (V.) braziliensis* (n=1) | CUTANEOUS |
|  | RIO DE JANEIRO or AMAZONAS (n=1) | *L. (V.) braziliensis* (n=1) | CUTANEOUS |
|  | RIO DE JANEIRO or AMAZONAS or MATO GROSSO (n=1) | *L. (V.) braziliensis* (n=1) | CUTANEOUS |
|  | RIO DE JANEIRO or AMAZONAS or MATO GROSSO (n=1) | *L. (V.) naiffi* (n=1) | CUTANEOUS |
|  | RIO DE JANEIRO or DISTRITO FEDERAL or SÃO PAULO (n=1) | *L. (V.) braziliensis* (n=1) | MUCOSAL |
|  | RIO DE JANEIRO or PARÁ (n=1) | *L. (V.) braziliensis* (n=1) | CUTANEOUS |
|  | RIO DE JANEIRO or PARAÍBA (n=1) | *L. (V.) braziliensis* (n=1) | MUCOSAL |
| BOLÍVIA  (n=1) | DEPARTAMENTO DE SANTA CRUZ (SANTA CRUZ DE LA SIERRA) | *L. (V.) braziliensis* (n=1) | MUCOSAL |
| ECUADOR  (n=1) | INDETERMINATE | *L. (V.) braziliensis* (n=1) | CUTANEOUS |
| FRENCH GUIANA (n=1) | INDETERMINATE | *L. (V.) guyanensis* (n=1) | MUCOCUTANEOUS |
