# Supplementary material for: Geospatial analysis of tegumentary leishmaniasis in Rio de Janeiro state, Brazil from 2000 to 2015: Species typing and flow of travelers and migrants with leishmaniasis
Source: PLoS Negl Trop Dis. 2019 Nov 15;13(11):e0007748. doi: 10.1371/journal.pntd.0007748 (PMC6857848; doi:10.1371/journal.pntd.0007748)
Supplement: S1 Consensus — (DOCX) [file pntd.0007748.s004.docx]

**Consensus Sequence of P4 – GenBank accession number MN508061**

5’TCTGGATCATTTTTCGATGATTACACCAAAAAACATACAACTCCGGGGAGGCTTGTGTTTTCTAGCAAGCCTTTCCCACAGATACGCAATACAATCTATATATGTATATATATAGACACAACATACAGTAGAAAAAGGCCGATCGACGTTAACATATCGCGTATACAACAAAAAAGTTCGTTCTACGGCTTTTTTTTTGGCGGCGTGCGGGGATAACGGCTCACATAACGTGTCGCGATGGATGACTTGGCTTCCTATTTCGTTGAAGAACGCAGAAAAGTGCGATAAGTGGTATCAAA3’
